# Full-Vectorial *3D* Microwave Imaging of Sparse Scatterers through a Multi-Task Bayesian Compressive Sensing Approach

**DOI:** 10.3390/jimaging5010019

**Published:** 2019-01-15

**Authors:** Marco Salucci, Lorenzo Poli, Giacomo Oliveri

**Affiliations:** 1 ELEDIA Research Center (ELEDIA@UniTN—University of Trento), Via Sommarive 9, I-38123 Trento, Italy; 2 ELEDIA Research Center (ELEDIA@L2S—UMR 8506), 3 rue Joliot Curie, 91192 Gif-sur-Yvette, France

**Keywords:** microwave imaging, inverse scattering, Bayesian compressive sensing (*BCS*), contrast source inversion (*CSI*), *3D*

## Abstract

In this paper, the full-vectorial three-dimensional (*3D*) microwave imaging (*MI*) of sparse scatterers is dealt with. Towards this end, the inverse scattering (*IS*) problem is formulated within the contrast source inversion (*CSI*) framework and it is aimed at retrieving the sparsest and most probable distribution of the contrast source within the imaged volume. A customized multi-task Bayesian compressive sensing (*MT-BCS*) method is used to yield regularized solutions of the *3D-IS* problem with a remarkable computational efficiency. Selected numerical results on representative benchmarks are presented and discussed to assess the effectiveness and the reliability of the proposed *MT-BCS* strategy in comparison with other competitive state-of-the-art approaches, as well.

## 1. Introduction

Microwave imaging (*MI*) techniques are aimed at inferring the complex permittivity distribution within an inaccessible investigation domain from the scattering interactions between the matter and probing electromagnetic (*EM*) waves [1]. They have been successfully applied in several diagnostic scenarios including non-destructive testing and evaluation [2,3], through-wall imaging [4], subsurface prospecting [5,6,7,8,9,10], and structural health monitoring [11]. Moreover, they represent a very appealing technology in many biomedical applications [12] such as, for instance, breast cancer detection [13,14,15,16,17,18,19,20,21,22] thanks to the use of non-ionizing radiations. To date, significant efforts have been mostly devoted to the development of two-dimensional (*2D*) *MI* algorithms, mainly based on transverse-magnetic (*TM*) [23,24,25,26,27,28] or transverse-electric (*TE*) polarized [29] configurations, rather than fully-vectorial three-dimensional (*3D*) ones [30]. As a matter of fact, under the assumption that the *EM* properties of the unknown scattering scenario are invariant along a longitudinal direction, the arising inverse scattering (*IS*) problem can be recast to the solution of simplified scalar Helmholtz equations [1]. On the other hand, *2D-MI* approaches are prone to errors when finite-volume scatterers are under test [31] because of the over-simplified modelling of the scattering phenomena. The slower evolution of *3D-MI* techniques has been mainly caused by the higher complexity of both data-collection/storage and image reconstruction processes with respect to the tomographic (*2D*) case. Moreover, a significantly larger number of unknowns has to be retrieved and it becomes very hard to manage when there is the need of high-resolution images (e.g., a realistic discretization of a human thorax needs millions of voxels for having a clinical significance [32]). Furthermore, the amount of non-redundant information on the investigation domain achievable from measurements is upper-bounded and the ratio between unknowns and scattering data turns out to be very high [33]. Owing to such limitations, solving *3D-MI* problems faces hard challenges and it requires the non-trivial implementation of effective countermeasures to both the *non-linearity* and the *ill-posedness* issues of the arising full-vectorial *IS* problem.

Dealing with *3D* scenarios, synthetic aperture radar (*SAR*)-based methodologies such as confocal *MI* [34] and synthetic near-field focusing [5] have been proposed. They are based on the emission of wide-band pulses from multiple transmitting positions and the successive processing of the collected echoes. Only target detection and localization (i.e., a *qualitative* imaging of the investigation domain) is typically yielded, while *quantitatively* retrieving the distribution of the *EM* properties needs the numerical solution of the non-linear scattering equations. Towards this end, effective *3D-MI* approaches have been presented in the scientific literature. They are based on the processing of time-domain [19,35] or frequency-domain [36,37] data and, due to the extremely-wide dimension of the solution space, which is usually proportional to the number of discretization domains, state-of-the-art methodologies are primarily based on deterministic methods (e.g., Gauss-Newton (*GN*) [36,38], Conjugate-Gradient (*CG*) [39], Level Set (*LS*) [16], and Inexact-Newton (*IN*) [40] methods, possibly formulated in different functional spaces [41]), even though they do not *a priori* guarantee to reach the actual solution (i.e., the global optimum of the cost function quantifying the mismatch between measured and estimated scattering data). To overcome such a drawback, either very efficient forward solvers [42,43] have been introduced or stochastic multi-agent inversion algorithms, suitably integrated with multi-resolution strategies [37] for a sustainable customization to the *3D* case, have been successfully exploited. Alternatively, computationally-efficient approaches to the *MI* problem have been recently explored within the compressive sensing (*CS*) framework [23,29,44,45,46,47,48,49]. Despite the early stage [47,50] of their implementation in *3D* cases, very interesting applicative examples are already available [20].

According to the *CS* theory, *sparseness priors* can be enforced to solve the *IS* problem and to yield a regularized solution provided that it admits a representation with few non-null coefficients in a suitably chosen basis [44,45]. However, since available *CS* solvers generally deal with linear problems, many sparsity-promoting approaches have been formulated within Born-like approximations, their success being limited to weak scatterers or to specific applications where a qualitative imaging is enough [29]. Alternatively, contrast source inversion (*CSI*)-based formulations of the *IS* problem can be successfully employed to yield accurate reconstructions also in the presence of scatterers with high *EM* contrast with respect to the surrounding medium [23]. Following such a line of reasoning, this paper is aimed at presenting a novel computationally-efficient approach, based on a Bayesian *CS* (*BCS*) method, to solve the *3D-IS* problem concerned with non-weak scatterers. Towards this end, the full-vectorial *IS* problem is formulated within a probabilistic *CSI* framework and then it is efficiently solved through a customized multi-task *BCS* (*MT-BCS*) strategy.

The outline of the paper is as follows. The formulation of the *3D-CSI MI* problem is detailed in Section 2, while the proposed *BCS*-based solution strategy is described in Section 3. Selected numerical results, from representative test cases, are presented and compared with competitive state-of-the-art alternatives in Section 4. Finally, some concluding remarks are drawn (Section 5).

## 2. Mathematical Formulation

**Figure 1:**
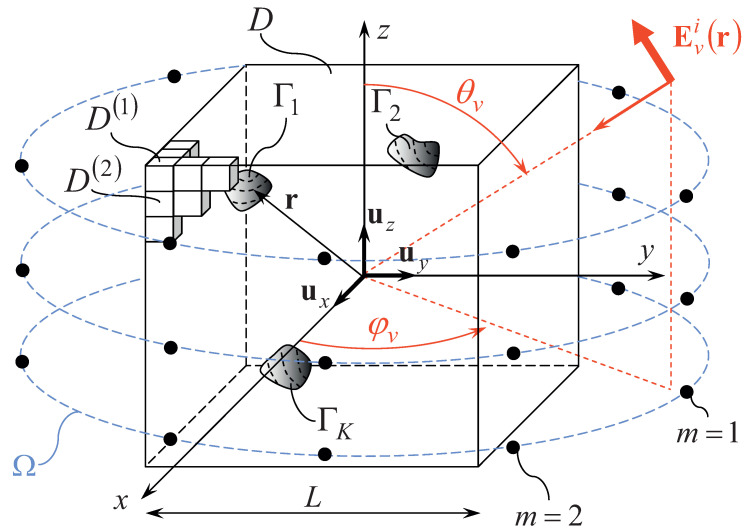
Geometry of the *3D-MI* microwave imaging problem.

Let us consider a *3D* isotropic non-magnetic [$\mu \left(r\right)=\mu_{0}$] scattering scenario characterized by a relative permittivity distribution, $\varepsilon \left(r\right)$, and a conductivity profile, $\sigma \left(r\right)$, r being the position vector defined as $r=xu_{x}+yu_{y}+zu_{z}$, $u_{p}$ being the unit vector along the *p*-th ($p=\left\{x,\;y,\;z\right\}$) direction. The goal of the *MI* problem at hand is to estimate the contrast function [51]

(1) $$\tau \left(r\right)≜\left[\varepsilon \left(r\right)-1\right]-j\frac{\sigma \left(r\right)-\sigma_{0}}{\omega \varepsilon_{0}}$$

within the investigation domain D (i.e., ∀r∈D), ω=2πf, $\varepsilon_{0}$, and $\sigma_{0}$ being the angular frequency (A time dependency factor $\exp\left(j\omega t\right)$ is assumed and omitted hereinafter to simplify the notation, but without loss of generality in the mathematical formulation.), the background permittivity and conductivity (with $\sigma_{0}=0$ hereinafter), respectively, and ≜ standing for “defined as”. Towards this end, a set of *V* monochromatic (*f* being the working frequency) plane-waves impinging from known angular directions $\left(\theta_{v},\;\varphi_{v}\right)$, v=1,…,V, with known electric field

(2) $$E_{v}^{i}\left(r\right)=\sum_{p=\left\{x,\;y,\;z\right\}}E_{v,p}^{i}\left(r\right)u_{p}\;v=1,\;\ldots ,\;V$$

is used to successively probe the unaccessible domain D ( Figure 1).

Under such hypotheses and by adopting a *CSI* formulation [52] for the scattering problem, the electromagnetic interactions between the scatterers in D and the *v*-th (v=1,…,V) incident wave can be mathematically described through the following integral *data* equation

(3) $$E_{v}^{s}\left(r\right)=-\omega^{2}\varepsilon_{0}\mu_{0}\int \int \int_{D}J_{v}\left(r^{\prime }\right)\cdot G\left(r,\;r^{\prime }\right)dr^{\prime }\;r\in \Omega$$

where Ω is an observation domain external to D (Ω∩D=∅—Figure 1), $E_{v}^{s}\left(r\right)$ [$E_{v}^{s}\left(r\right)=\sum_{p=\left\{x,\;y,\;z\right\}}E_{v,p}^{s}\left(r\right)u_{p}$ ] is the *v*-th (v=1,…,V) *scattered* field defined as the difference between the *v*-th (v=1,…,V) electric field with, $E_{v}\left(r\right)=\sum_{p=\left\{x,\;y,\;z\right\}}E_{v,p}\left(r\right)u_{p}$ (i.e., the *v*-th *total* electric field), and without, $E_{v}^{i}\left(r\right)$ (i.e., the *v*-th *incident* electric field), the scatterers in the background medium ($E_{v}^{s}\left(r\right)≜\left[E_{v}\left(r\right)-E_{v}^{i}\left(r\right)\right]$), · stands for the scalar product, and

(4) $$G\left(r,\;r^{\prime }\right)=\sum_{q=\left\{x,\;y,\;z\right\}}\sum_{p=\left\{x,\;y,\;z\right\}}G_{pq}u_{p}u_{q}=\frac{1}{4\pi }\left(I+\frac{1}{\omega^{2}\varepsilon_{0}\mu_{0}}▽▽\right)\frac{\exp\left(-j\omega \sqrt{\varepsilon_{0}\mu_{0}}\left|r-r^{\prime }\right|\right)}{\left|r-r^{\prime }\right|}$$

is the dyadic Green’s function for the homogeneous free-space background medium of dielectric and magnetic properties $\varepsilon_{0}$ and $\mu_{0}$, respectively, I being the unit tensor. In (3), $J_{v}$ [$J_{v}\left(r\right)=\sum_{p=\left\{x,\;y,\;z\right\}}J_{v,p}\left(r\right)u_{p}$] is the *v*-th (v=1,…,V) unknown contrast current density induced in the investigation domain (r∈D) by the *v*-th probing field (i.e., the *v*-th illumination of D)

(5) $$J_{v}\left(r\right)=\tau \left(r\right)\sum_{p=\left\{x,\;y,\;z\right\}}E_{v,p}\left(r\right)u_{p}$$

that models the scattering profile of D and it is defined as the *v*-th (v=1,…,V) *equivalent* source radiating in the background medium an electromagnetic field equal to the *v*-th (v=1,…,V) *scattered* field $E_{v}^{s}\left(r\right)$. Furthermore, the *v*-th (v=1,…,V) incident field $E_{v}^{i}\left(r\right)$ complies with the so-called integral *state* equation

(6) $$E_{v}^{i}\left(r\right)=E_{v}\left(r\right)+\omega^{2}\varepsilon_{0}\mu_{0}\int \int \int_{D}J_{v}\left(r^{\prime }\right)\cdot G\left(r,\;r^{\prime }\right)dr^{\prime }$$

within the investigation domain (r∈D).

To numerically deal with the inverse problem at hand, a set of *N* *3D* rectangular pulse functions

(7) $$\Psi^{\left(n\right)}\left(r\right)=\left\{\begin{array}{cc} 1 & \mathrm{if}\;r\in D^{\left(n\right)}\\ 0 & \mathrm{otherwise} \end{array}\right.;\;n=1,\;\ldots ,\;N$$

defined in a set of *N* cubic (also called *voxels*) sub-domains ($D=\cup_{n=1}^{N}D^{\left(n\right)}$—Figure 1) is adopted for yielding the following piece-wise constant representation of the *v*-th (v=1,…,V) unknown contrast source

(8) $$J_{v}\left(r\right)=\sum_{p=\left\{x,\;y,\;z\right\}}\sum_{n=1}^{N}J_{v,p}^{\left(n\right)}\Psi^{\left(n\right)}\left(r\right)u_{p}$$

where $J_{v,p}^{\left(n\right)}=J_{v,p}\left(r_{n}\right)$ ($p=\left\{x,\;y,\;z\right\}$) and $r_{n}$ denotes the barycentre of the *n*-th (n=1,…,N) voxel $D^{\left(n\right)}$.

By sampling the scattered field in *M* probing locations of Ω ($r_{m}\in \Omega$, m=1,…,M), it is possible to rewrite (3) in the following discrete form [1]

(9) $$E_{v}^{s}\left(r_{m}\right)=\sum_{p=\left\{x,\;y,\;z\right\}}\sum_{q=\left\{x,\;y,\;z\right\}}\sum_{n=1}^{N}J_{v,q}^{\left(n\right)}G_{pq}^{\left(mn\right)}u_{p},$$

where $G_{pq}^{\left(mn\right)}=-\omega^{2}\varepsilon_{0}\mu_{0}\int \int \int_{D^{\left(n\right)}}G_{pq}\left(r_{m},\;r^{\prime }\right)dr^{\prime }$ ($p,\;q=\left\{x,\;y,\;z\right\}$), or in compact matrix notation as follows

(10) $${\left[{\underline{E}}_{v,x}^{s},\;{\underline{E}}_{v,y}^{s},\;{\underline{E}}_{v,z}^{s}\right]}^{T}={\underline{\underline{G}}}^{ext}{\left[{\underline{J}}_{v,x},\;{\underline{J}}_{v,y},\;{\underline{J}}_{v,z}\right]}^{T}$$

where $.^{T}$ indicates the transpose operator, ${\underline{E}}_{v,p}^{s}≜\left[E_{v,p}^{s}\left(r_{m}\right);\;m=1,\;\ldots ,\;M\right]$ (${\underline{E}}_{v,p}^{s}\in C^{1\times M}$), ${\underline{J}}_{v,p}≜\left[J_{v,p}^{\left(n\right)};\;n=1,\;\ldots ,\;N\right]$ (${\underline{J}}_{v,p}\in C^{1\times N}$), $p=\left\{x,\;y,\;z\right\}$, and

(11) $${\underline{\underline{G}}}^{ext}≜\left[\begin{array}{ccc} {\underline{\underline{G}}}_{xx}^{ext} & {\underline{\underline{G}}}_{xy}^{ext} & {\underline{\underline{G}}}_{xz}^{ext}\\ {\underline{\underline{G}}}_{yx}^{ext} & {\underline{\underline{G}}}_{yy}^{ext} & {\underline{\underline{G}}}_{yz}^{ext}\\ {\underline{\underline{G}}}_{zx}^{ext} & {\underline{\underline{G}}}_{zy}^{ext} & {\underline{\underline{G}}}_{zz}^{ext} \end{array}\right]$$

is the external *3D* Green’s operator (${\underline{\underline{G}}}^{ext}\in C^{3M\times 3N}$) where the $\left(m,\;n\right)$-th (m=1,…,M; n=1,…,N) entry of the $\left(p,\;q\right)$-th ($p,\;q=\left\{x,\;y,\;z\right\}$) sub-matrix, ${\underline{\underline{G}}}_{pq}^{ext}\in C^{M\times N}$, is equal to $G_{pq}^{\left(mn\right)}$.

Under the hypothesis that the contrast distribution in D is *sparse* with respect to the basis at hand (7), it also turns out that the *v*-th (v=1,…,V) contrast source (8) can be numerically modeled with just 3×O (O≪N) non-zero coefficients $\left\{\left[J_{v,x}^{\left(o\right)};\;J_{v,y}^{\left(o\right)};\;J_{v,z}^{\left(o\right)}\right];\;o=1,\;\ldots ,\;O\right\}$, *O* being the number of sub-domains $D^{\left(o\right)}\in D$, o=1,…,O, occupied by scatterers (i.e., dielectric discontinuities of the background medium within the investigation domain).

## 3. Inversion Method

The matrix relation in (10) is representative of a severely *ill-posed* problem being (*i*) *ill-defined* since its solution is not-unique due to the presence of non-radiating sources in D that afford a null/not-measurable field in Ω and (*ii*) *ill-conditioned* since the condition number of ${\underline{\underline{G}}}^{ext}$, η, is large (η≫1). To counteract such a negative feature for yielding stable reconstructions of the *EM* properties of the investigation domain from the solution of (10) when processing noisy data, as well, enforcing the *a-priori* knowledge of the *sparseness* of the unknown equivalent source with respect to a suitable basis turns out to be an effective regularization strategy. Towards this end, the *CS* formulation based on the Bayesian theory is adopted to re-formulate the *3D-CSI* problem at hand in a *probabilistic* sense. Such a choice is done to avoid the need of numerically checking the fulfillment of the restricted isometry property (*RIP*) of the *3D* Green’s operator (11) as required by deterministic *CS* solvers. Indeed, whether such a compliance test is already very hard from a computational viewpoint for small-scale problems, it becomes computationally unfeasible in *3D* scattering scenarios since exponentially heavier than for *2D* cases.

In order to apply computationally-efficient *BCS* solvers, (10) is first rewritten as a larger real-valued linear problem

(12) $${\underline{E}}_{v,p}={\underline{\underline{G}}}_{p}\;{\underline{J}}_{v}$$

where ${\underline{E}}_{v,p}≜{\left[R\left({\underline{E}}_{v,p}^{s}\right),\;I\left({\underline{E}}_{v,p}^{s}\right)\right]}^{T}$ and ${\underline{J}}_{v}≜{\left[R\left({\underline{J}}_{v,p};\;p=\left\{x,\;y,\;z\right\}\right),\;I\left({\underline{J}}_{v,p};\;p=\left\{x,\;y,\;z\right\}\right)\right]}^{T}$ are the *p*-th ($p=\left\{x,\;y,\;z\right\}$) data vector, ${\underline{E}}_{v,p}\in R^{2M\times 1}$, and the *v*-th (v=1,…,V) unknown source vector, ${\underline{J}}_{v}\in R^{6N\times 1}$, respectively, while the Green’s matrix operator, ${\underline{\underline{G}}}_{p}\in R^{2M\times 6N}$, is given by

(13) $${\underline{\underline{G}}}_{p}≜\left[\begin{array}{cc} R\left(\begin{array}{ccc} {\underline{\underline{G}}}_{px}^{ext} & {\underline{\underline{G}}}_{py}^{ext} & {\underline{\underline{G}}}_{pz}^{ext} \end{array}\right) & -I\left(\begin{array}{ccc} {\underline{\underline{G}}}_{px}^{ext} & {\underline{\underline{G}}}_{py}^{ext} & {\underline{\underline{G}}}_{pz}^{ext} \end{array}\right)\\ I\left(\begin{array}{ccc} {\underline{\underline{G}}}_{px}^{ext} & {\underline{\underline{G}}}_{py}^{ext} & {\underline{\underline{G}}}_{pz}^{ext} \end{array}\right) & R\left(\begin{array}{ccc} {\underline{\underline{G}}}_{px}^{ext} & {\underline{\underline{G}}}_{py}^{ext} & {\underline{\underline{G}}}_{pz}^{ext} \end{array}\right) \end{array}\right]$$

$R\left(.\right)$ and $I\left(.\right)$ being the real and the imaginary part function, respectively. According to such a description, the original inverse scattering problem can be then formulated as follows

***3D-CSI BCS-Based Problem Formulation***—Starting from the measurement of ${\underline{E}}_{v,p}$ (v=1,…,V, $p=\left\{x,\;y,\;z\right\}$), and the knowledge of ${\underline{\underline{G}}}_{p}$ ($p=\left\{x,\;y,\;z\right\}$), determine the *sparsest* guess of ${\underline{J}}_{v}$, ${\underline{\hat{J}}}_{v}=\left\{{\hat{J}}_{v}^{\left(t\right)};\;t=1,\;\ldots ,\;6\times N\right\}$ as the maximum *a-posteriori* probability (*MAP*) estimate

(14) $${\underline{\hat{J}}}_{v}=\arg\left\{\max_{{\underline{J}}_{v}}\left[P\left(\left.{\underline{J}}_{v}\right|{\underline{E}}_{v,p}\right)\right]\right\}$$

provided that the support of ${\underline{\hat{J}}}_{v}$, $S_{v}=\left\{\left.t\in \left[1,\;\ldots ,\;6\times N\right]\right|{\hat{J}}_{v}^{\left(t\right)}\neq 0\right\}$ (v=1,…,V) is the same for all *V* different illuminations (i.e., $S_{1}=S_{2}=\ldots =S_{V}$).

In order to enforce the physical correlation existing among the *V* solutions of (14) as well as between the unknown contrast sources and the vectorial components of the known scattered field, a customized version of the *MT-BCS* solver [53] is adopted by setting the number of parallel “tasks” to $H=\left(3\times V\right)$ (owing to the 3D nature of the problem). More in detail, (14) is firstly reformulated in the Bayesian *MT* framework as [29,53]

(15) $${\underline{\hat{J}}}_{v}=\arg\left\{\max_{{\underline{J}}_{v}}\left[\frac{P\left(\left.{\underline{E}}_{v,p}\right|{\underline{J}}_{v}\right)P\left({\underline{J}}_{v}\right)}{P\left({\underline{E}}_{v,p}\right)}\right]\right\}$$

starting from the definition of a shared prior $P\left({\underline{J}}_{v}\right)$ that statistically links the 3×V *parallel* problem unknowns as [29,53]

(16) $$P\left({\underline{J}}_{v}\right)=\int P\left(\left.{\underline{J}}_{v}\right|\underline{\psi }\right)d\underline{\psi }$$

where $\underline{\psi }$ ($\underline{\psi }≜\left\{\psi^{\left(t\right)};\;t=1,\;\ldots ,\;6\times N\right\}$) is the set of *shared*—among the *V*-views and the three Cartesian scattered field components—hyper-parameters. By assuming a hierarchical Gaussian model for $P\left(\left.{\underline{J}}_{v}\right|\underline{\psi }\right)$ and substituting (16) in (15) [29,53], the *MT-BCS* *v*-th (v=1,…,V) source term is then given after simple manipulations [29] by the following expression

(17) $${\underline{\hat{J}}}_{v}=\frac{1}{3}\sum_{p=\left\{x,\;y,\;z\right\}}{\left({\underline{\underline{G}}}_{p}^{T}{\underline{\underline{G}}}_{p}+\underline{\underline{A}}\right)}^{-1}{\underline{\underline{G}}}_{p}^{T}{\underline{E}}_{v,p}$$

where $\underline{\underline{A}}=diag\left(\underline{\hat{\psi }}\right)$ and $\underline{\hat{\psi }}≜\left\{{\hat{\psi }}^{\left(t\right)};\;t=1,\;\ldots ,\;6\times N\right\}$. These latter are determined with a computationally-efficient relevant vector machine (*RVM*) [54] by solving the following maximum marginal likelihood problem

(18) $$\underline{\hat{\psi }}=\arg\left\{max_{\underline{\psi }}\left[L\left(\underline{\psi }\left|\alpha ,\;\beta \right.\right)\right]\right\}$$

$L\left(\underline{\psi }\left|\alpha ,\;\beta \right.\right)$ being the logarithmic marginal likelihood function for the fully-vectorial problem given by

(19) $$\begin{array}{c} L\left(\underline{\psi }\left|\alpha ,\;\beta \right.\right)=-\frac{1}{2}\sum_{v=1}^{V}\sum_{p=\left\{x,\;y,\;z\right\}}\left\{2\left(M+\alpha \right)\log\left[{\underline{E}}_{v,p}^{T}\right.\right.\\ \left.\left.{\left(\underline{\underline{I}}+{\underline{\underline{G}}}_{p}{\underline{\underline{A}}}^{-1}{\underline{\underline{G}}}_{p}^{T}\right)}^{-1}{\underline{E}}_{v,p}+2\beta \right]+\log\left|\underline{\underline{I}}+{\underline{\underline{G}}}_{p}{\underline{\underline{A}}}^{-1}{\underline{\underline{G}}}_{p}^{T}\right|\right\} \end{array}$$

where $\underline{\underline{I}}$ is the identity matrix, while α and β are user-defined control parameters. Once ${\underline{\hat{J}}}_{v}$ (v=1,…,V) has been estimated through (17), the contrast function, $\hat{\tau }\left(r\right)=\sum_{n=1}^{N}{\hat{\tau }}^{\left(n\right)}\Psi^{\left(n\right)}\left(r\right)$, is retrieved by computing the corresponding expansion coefficient vector $\underline{\hat{\tau }}=\left\{{\hat{\tau }}^{\left(n\right)}\in C;\;n=1,\;\ldots ,\;N\right\}$, whose *n*-th (n=1,…,N) entry is given by

(20) $${\hat{\tau }}^{\left(n\right)}=\sum_{p=\left\{x,\;y,\;z\right\}}\sum_{v=1}^{V}\frac{{\hat{J}}_{v,p}^{\left(n\right)}}{3\times V\times {\hat{E}}_{v,p}\left(r_{n}\right)}$$

where ${\hat{E}}_{v,p}\left(r_{n}\right)$ is the *p*-th ($p=\left\{x,\;y,\;z\right\}$) component of the *v*-th (v=1,…,V) total field in the *n*-th voxel ($r_{n}\in D^{\left(n\right)}$) (n=1,…,N) yielded from the following field relation

(21) $${\hat{E}}_{v,p}\left(r_{n}\right)=E_{v,p}^{i}\left(r_{n}\right)-\omega^{2}\varepsilon_{0}\mu_{0}\sum_{q=\left\{x,\;y,\;z\right\}}\sum_{n=1}^{N}{\hat{J}}_{v,q}^{\left(n\right)}\int \int \int_{D^{\left(n\right)}}G_{pq}\left(r_{n},\;r^{\prime }\right)dr^{\prime },$$

while the coefficient ${\hat{J}}_{v,p}^{\left(n\right)}$ (n=1,…,N; v=1,…,V; $p=\left\{x,\;y,\;z\right\}$) is derived from ${\underline{\hat{J}}}_{v}$ (17) according to the following mapping

(22) $${\hat{J}}_{v,p}^{\left(n\right)}=\left\{\begin{array}{cc} {\hat{J}}_{v}^{\left(n\right)}+j{\hat{J}}_{v}^{\left(n+3\times N\right)} & \mathrm{if}\;p=x\\ {\hat{J}}_{v}^{\left(n+N\right)}+j{\hat{J}}_{v}^{\left(n+4\times N\right)} & \mathrm{if}\;p=y\\ {\hat{J}}_{v}^{\left(n+2N\right)}+j{\hat{J}}_{v}^{\left(n+5\times N\right)} & \mathrm{if}\;p=z \end{array}\right..$$

## 4. Numerical Assessment

In this Section, representative results from a wide numerical analysis are presented and discussed to assess the reconstruction capabilities and the robustness of the proposed *3D-MI* approach. Moreover, some practical guidelines for the optimal setting of its key calibration parameters will be provided for helping the interested users. Towards this end, the following reference scenario has been considered. A cubic volume of side L=1.25 [λ], λ being the free-space wavelength, has been chosen as investigation domain D and it has been probed by V=48 plane-waves impinging from the *V* angular directions $\left(\theta_{v},\;\varphi_{v}\right)=\left(\frac{\pi }{2},\;\left(v-1\right)\frac{2\pi }{V}\right)$, v=1,…,V. The scattered field has been collected in M=48 locations

(23) $$\left\{\begin{array}{c} x_{m}=\rho \cos\left[\left(m+1-\kappa_{m}\frac{M}{3}\right)\frac{6\pi }{M}\right]\\ y_{m}=\rho \sin\left[\left(m+1-\kappa_{m}\frac{M}{3}\right)\frac{6\pi }{M}\right]\\ z_{m}=\frac{L}{2}\left(\kappa_{m}-1\right) \end{array}\right.;\;m=1,\;\ldots ,\;M$$

where $\kappa_{m}=\left\lfloor \frac{3\left(m-1\right)}{M}\right\rfloor$ ($\left\lfloor \cdot \right\rfloor$ being the *floor* operator) and ρ=3.2 [λ]. To avoid the so-called *inverse crime* [1], two different voxel-based discretization of D have been considered for the inverse (N=10×10×10) and the forward ($N_{fwd}=20\times 20\times 20$) problem, respectively. As for a quantitative evaluation of the reconstructions, the following error metric has been computed

(24) $$\xi_{R}≜\frac{1}{N_{R}}\sum_{n=1}^{N_{R}}\frac{\left|{\hat{\tau }}^{\left(n\right)}-\tau^{\left(n\right)}\right|}{\left|\tau^{\left(n\right)}\right|+1}$$

for the whole investigation domain (R=tot ⇒ $N_{tot}=N$), the scatterers support (R=int ⇒ $N_{int}=O$), and its complementary region (It is worthwhile to remark that the scatterer support is not an *a-priori* information exploited during the inversion, but it is rather employed in the post-processing phase only to compute the error metrics (24).) [R=ext ⇒ $N_{ext}=\left(N-O\right)$], $\tau^{\left(n\right)}$ and ${\hat{\tau }}^{\left(n\right)}$ being the actual and the estimated contrast of the *n*-th (n=1,…,N) voxel in D, respectively.

**Figure 2:**
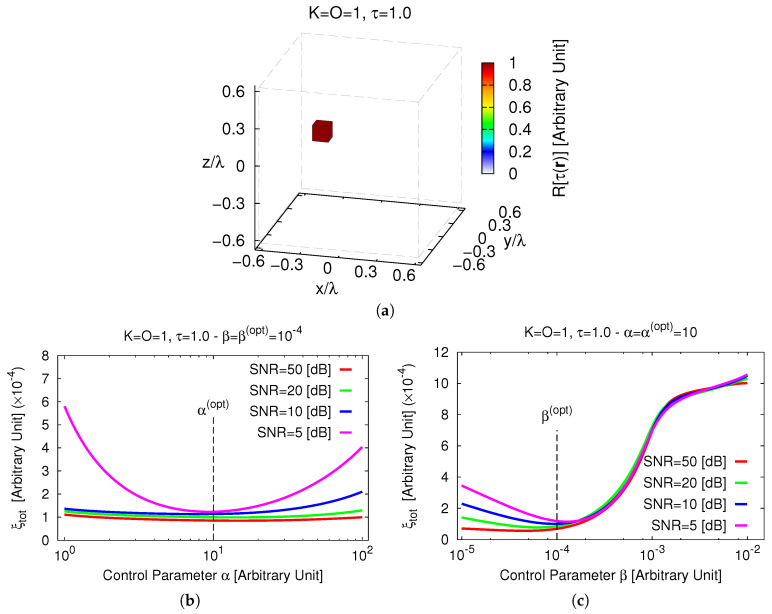
*Sensitivity Analysis* (K=1, O=1, τ=1.0, $SNR\in \left[5,\;50\right]$ [dB])—Actual contrast function (**a**). Behavior of the total error, $\xi_{tot}$, versus the *MT-BCS* control parameters: (**b**) $\alpha \in \left[1,\;10^{2}\right]$ ($\beta =\beta^{\left(opt\right)}=10^{-4}$) and (**c**) $\beta \in \left[10^{-5},\;10^{-2}\right]$ ($\alpha =\alpha^{\left(opt\right)}=10$).

The first test case is concerned with the retrieval of a single (K=1, *K* being the number of disconnected scattering regions lying in D) off-centered scatterer composed by a single-voxel (O=1) of side $\ell_{x}=\ell_{y}=\ell_{z}=0.125$ [λ] with contrast τ=1.0 (Figure 2a). First, a sensitivity analysis for setting the optimal trade-off values of the control parameters of the *MT-BCS* solver [i.e., α and β in (19)] has been carried out. More specifically, the reconstruction error (24) has been computed by varying the values of the calibration coefficients within the ranges $\alpha \in \left[1,\;10^{2}\right]$ and $\beta \in \left[10^{-5},\;10^{-2}\right]$ for different signal-to-noise ratios (*SNR*s). Figure 2b shows that the reconstruction “quality” is almost insensitive to the choice of α when SNR>5 [dB] and it mainly depends on the noise level.

**Figure 3:**
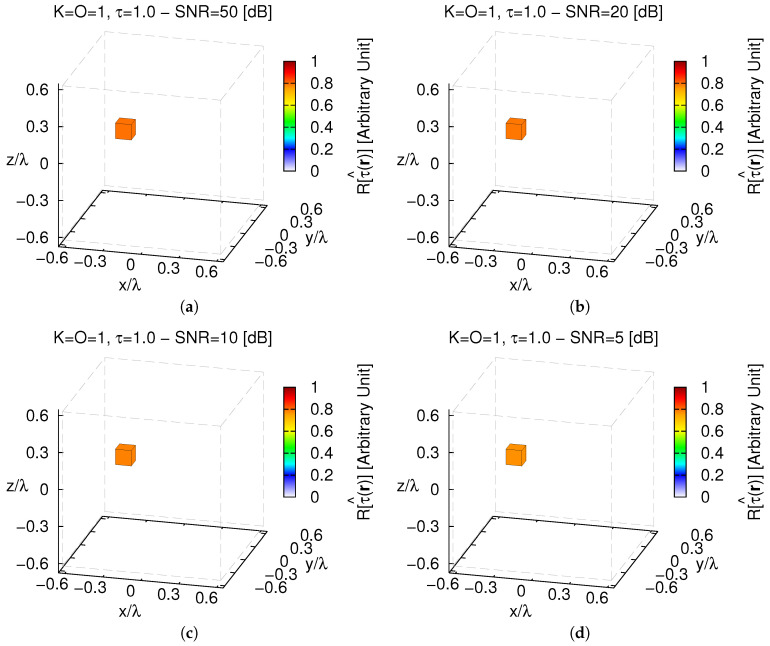
*Numerical Assessment* (K=1, O=1, τ=1.0)—*MT-BCS* reconstructions when processing the scattering data with (**a**) SNR=50 [dB] ($\hat{\tau }=0.81$); (**b**) SNR=20 [dB] ($\hat{\tau }=0.79$); (**c**) SNR=10 [dB] ($\hat{\tau }=0.78$), and (**d**) SNR=5 [dB] ($\hat{\tau }=0.77$).

Differently, significant degradations occur when letting $\beta >5\times 10^{-4}$ whatever the *SNR* (Figure 2c). By computing the optimal *trade-off* value as $\varsigma^{\left(opt\right)}≜\frac{\int_{SNR}{\left.\varsigma^{\left(opt\right)}\right\rfloor }_{SNR}dSNR}{\int_{SNR}dSNR}$, ${\left.\varsigma^{\left(opt\right)}\right\rfloor }_{SNR}≜\arg\left\{\min_{\varsigma }\left(\left.\xi_{tot}\right|SNR\right)\right\}$ being the best value of $\varsigma =\left\{\alpha ,\;\beta \right\}$ at a fixed *SNR* ($\left.\cdot \right\rfloor$ standing for “evaluated at”), it turned out that $\alpha^{\left(opt\right)}=10$ and $\beta^{\left(opt\right)}=10^{-4}$. These thresholds have then been used throughout the whole numerical validation. A pictorial view of the *3D MT-BCS* reconstructions when processing different noisy data is shown in Figure 3.

As it can be observed, the object support is always faithfully detected regardless of the amount of noise. There are only slight deviations from the actual contrast value (e.g., ${\left.\hat{\tau }\right\rfloor }_{SNR=50\;[\mathrm{dB}]}=0.81$—Figure 3a; ${\left.\hat{\tau }\right\rfloor }_{SNR=5\;[\mathrm{dB}]}=0.77$—Figure 3d) as quantitatively indicated by the corresponding values of the error index $\xi_{tot}$ being equal to ${\left.\xi_{tot}\right\rfloor }_{SNR=50\;[\mathrm{dB}]}=9.58\times 10^{-5}$ (Figure 3a) and ${\left.\xi_{tot}\right\rfloor }_{SNR=5\;[\mathrm{dB}]}=1.16\times 10^{-4}$ (Figure 3d) at the highest and lowest *SNR*s, respectively.

In order to prove that such results are not due to a customization of the *MT-BCS* solver to the specific scenario at hand, a set of W=100 inversions has been performed by randomly changing the position of the same target within the investigation domain. The results of such a statistical analysis are summarized in Table 1 where the minimum ($\xi_{tot}^{\min}≜\min_{w=1,\;\ldots ,\;W}\left\{\xi_{tot,w}\right\}$), the maximum ($\xi_{tot}^{\max}≜\max_{w=1,\;\ldots ,\;W}\left\{\xi_{tot,w}\right\}$), the average ($\xi_{tot}^{\mathrm{avg}}≜\frac{1}{W}\sum_{w=1}^{W}\xi_{tot,w}$), and the variance [$\xi_{tot}^{\mathrm{var}}≜\frac{1}{W}\sum_{w=1}^{W}{\left(\xi_{tot,w}-\xi_{tot}^{\mathrm{avg}}\right)}^{2}$] values of the total error among the *W* experiments are reported. Whatever the *SNR*, the scatterer retrieval is very accurate since on average $\xi_{tot}^{\mathrm{avg}}<1.0\times 10^{-4}$ (Table 1) and the variance of the reconstruction error, $\xi_{tot}^{\mathrm{var}}$, is very small (i.e., $\xi_{tot}^{\mathrm{var}}<5.0\times 10^{-11}$—Table 1).

**Figure 4:**
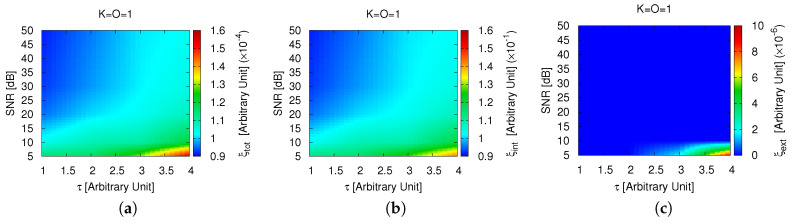
*Numerical Assessment* (K=1, O=1, $\tau \in \left[1.0,\;4.0\right]$, $SNR\in \left[5,\;50\right]$ [dB])—Behavior of the (**a**) total ($\xi_{tot}$); (**b**) internal ($\xi_{int}$); and (**c**) external ($\xi_{ext}$) reconstruction errors when processing the scattering data with the *MT-BCS*.

Such a positive outcome holds true when dealing with stronger scatterers, as well. Thanks to the *CSI* formulation, which avoids any physical assumption/approximation in the application of the *CS* to the *3D* scattering equations (Section 2), faithful data inversions are yielded also when increasing the actual contrast well-beyond the value of the first example. As a proof, the plots of $\xi_{tot}$ (Figure 4a), $\xi_{int}$ (Figure 4b), and $\xi_{ext}$ (Figure 4c) versus τ and the noise level indicate that D can be carefully imaged when the scatterer is at least up to τ=4.0.

**Figure 5:**
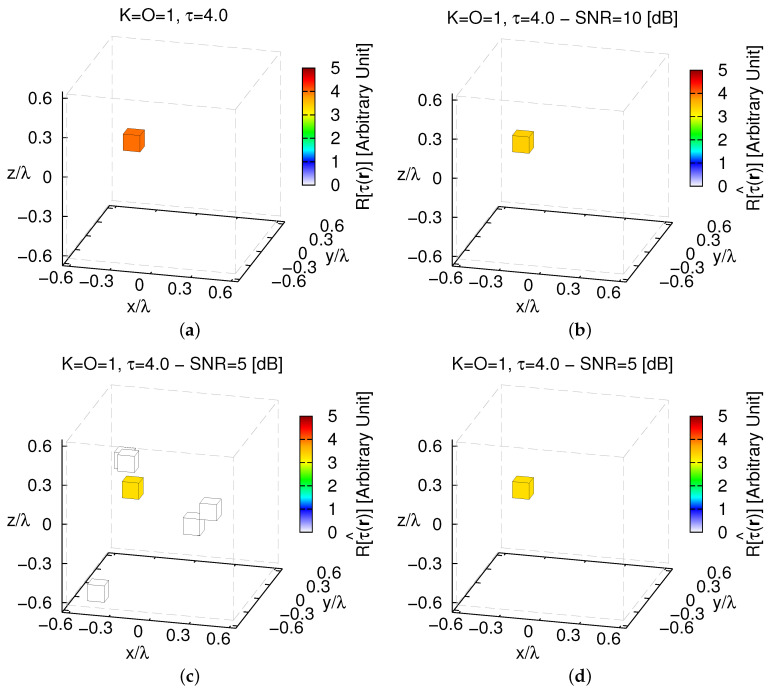
*Numerical Assessment* (K=1, O=1, τ=4.0)—(**a**) Actual contrast function and (**b**,**c**) *MT-BCS* reconstructions when processing the scattering data with (**b**) SNR=10 [dB] ($\hat{\tau }=3.40$), (**c**) SNR=5 [dB] (unfiltered) ($\hat{\tau }=3.28$), and (**d**) SNR=5 [dB] (filtered $\tau_{th}=10^{-3}$) ($\hat{\tau }=3.28$).

As expected, the reconstruction progressively degrades when higher and higher contrasts are at hand especially when processing highly blurred data. Figure 5 shows the actual and the retrieved volumetric distributions when τ=4.0 and SNR≤10 [dB]. As it can be inferred, some artifacts are present only in very harsh conditions (Figure 5c—SNR=5 [dB]) when the external error (Figure 4c) grows from ${\left.\xi_{ext}\right\rfloor }_{SNR=5\;[\mathrm{dB}]}^{\tau =1.0}=0.0$ (Figure 3d) to ${\left.\xi_{ext}\right\rfloor }_{SNR=5\;[\mathrm{dB}]}^{\tau =4.0}=8.96\times 10^{-6}$ (Figure 5c). Nevertheless, it is worth pointing out that these inaccuracies correspond to voxels with a very low contrast (i.e., $\hat{\tau }<3\times 10^{-4}$—Figure 5c) that can be easily filtered out by a simple (i.e., the result is not so sensitive to the choice of the threshold value $\tau_{th}$) thresholding (Figure 5d—$\tau_{th}=10^{-3}$) and the scatterer is always correctly localized (Figure 5c as well as Figure 5d vs. Figure 5a).

**Figure 6:**
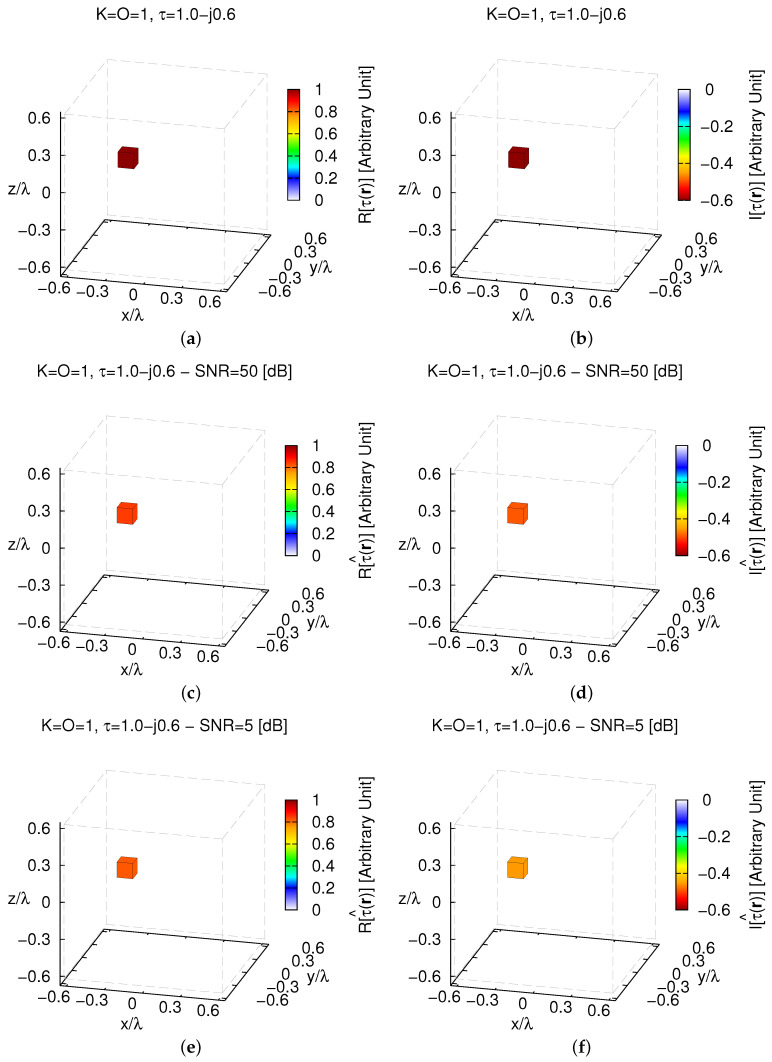
*Numerical Assessment* (K=1, O=1, τ=1.0−0.6j)—Real (**a**,**c**,**e**) and imaginary parts (**b**,**d**,**f**) of the (**a**,**b**) actual contrast function and of the (**b**–**f**) *MT-BCS* reconstructed profiles when processing the scattering data with (**c**,**d**) SNR=50 [dB] ($\hat{\tau }=0.84-0.49j$), (**e**,**f**) SNR=5 [dB] ($\hat{\tau }=0.81-0.45j$).

The previous examples have dealt with real-valued target contrasts. However, the imaginary part of τ is not enforced to be zero during the inversion (see Section 3). In order to assess the reliability of the *MT-BCS* also when complex contrast values are at hand, the retrieval of a K=1, O=1 target with τ=1.0−0.6j (Figure 6a,b) has been considered next. The analysis of the retrieved profiles (Figure 6) shows that the location, size, and contrast of the scatterer is correctly retrieved by the proposed imaging strategy both in low (e.g., SNR=50 [dB]—Figure 6c,d) and in high noise conditions (e.g., SNR=5 [dB]—Figure 6e,f), as shown by the corresponding error indexes (e.g., ${\left.\xi_{tot}\right\rfloor }_{SNR=50\;[\mathrm{dB}]}=8.63\times 10^{-5}$—Figure 6c,d; ${\left.\xi_{tot}\right\rfloor }_{SNR=5\;[\mathrm{dB}]}=1.06\times 10^{-4}$—Figure 6e,f).

**Figure 7:**
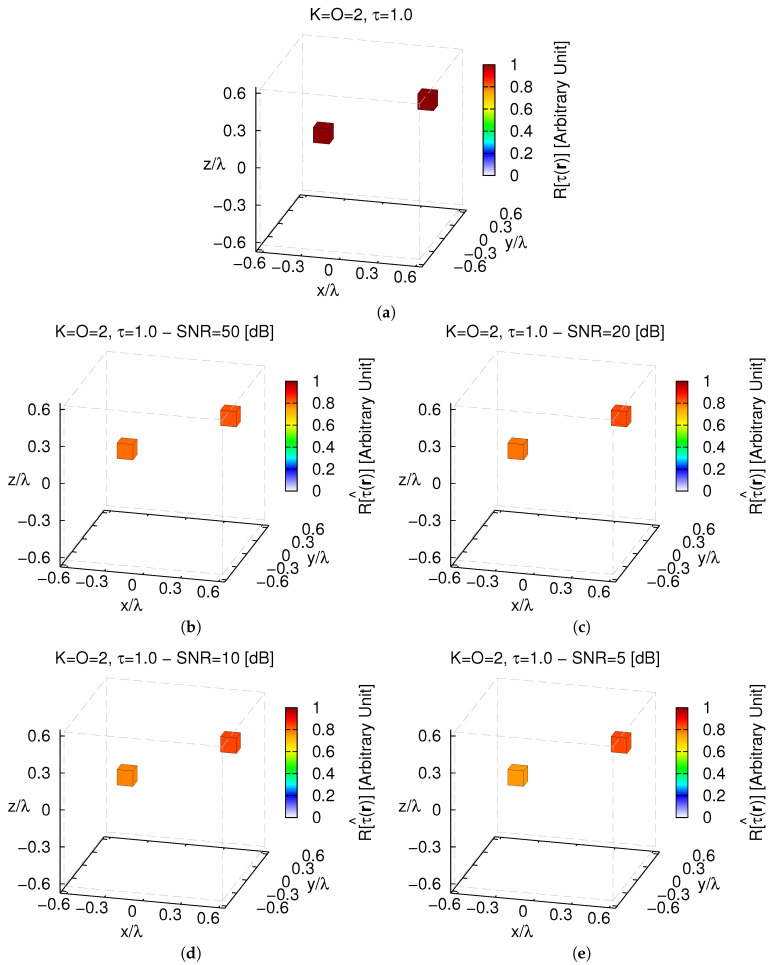
*Numerical Assessment* (K=2, O=2, τ=1.0)—(**a**) Actual contrast function and *MT-BCS* reconstructions when processing the scattering data with (**b**) SNR=50 [dB] (${\hat{\tau }}_{\max}=0.82$), (**c**) SNR=20 [dB] (${\hat{\tau }}_{\max}=0.84$), (**d**) SNR=10 [dB] (${\hat{\tau }}_{\max}=0.84$), and (**e**) SNR=5 [dB] (${\hat{\tau }}_{\max}=0.83$).

Being assessed the effectiveness of the proposed approach in reconstructing the sparsest (O=1) actual profile, let us now analyze its performance for scenarios exhibiting a lower *sparsity* order with respect to the selected voxel basis (7). Towards this end, a second single-voxel scatterer has been added to the configuration of the first test case (Figure 2a), but in a different position in D (K=O=2, τ=1.0—Figure 7a).

As it can be inferred from the plots in Figure 7b–e, the *MT-BCS* is always able to correctly retrieve both scatterers even from very low-noise data (e.g., ${\left.\xi_{tot}\right\rfloor }_{SNR=5\;[\mathrm{dB}]}=9.80\times 10^{-4}$—Figure 7e). However, the same statistical analysis of the single-scatterer/single-voxel case (i.e., randomly changing the locations of the scatterers) here results in wider variations of the integral error (i.e., $\xi_{tot}^{\mathrm{var}}\geq 4.5\times 10^{-7}$ (Table 2) vs. $\xi_{tot}^{\mathrm{var}}\geq 1.11\times 10^{-11}$ (Table 1)). Therefore, to better understand how the inversion accuracy is affected by the positions of the scatterers within the investigation domain, a further set of experiments has been performed by deterministically changing the relative locations of the K=2 scatterers, *d* being the distance of each one of them from the origin.

**Figure 8:**
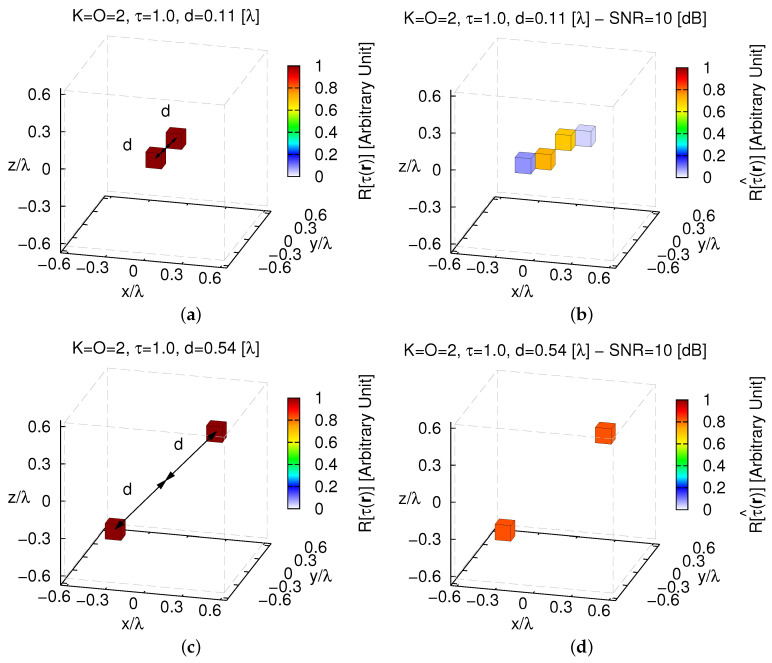

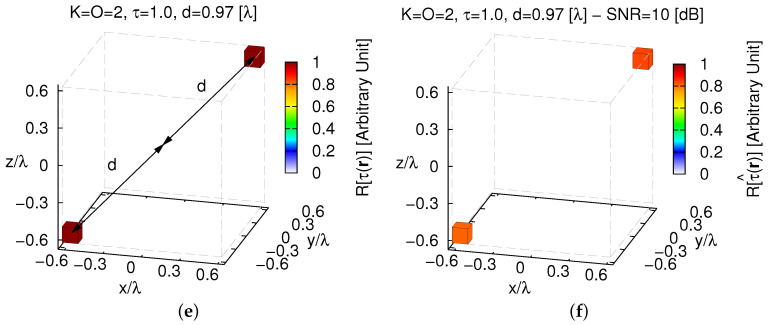
*Numerical Assessment* (K=2, O=2, τ=1.0, SNR=10 [dB])—(**a**,**c**,**e**) Actual contrast function and (**b**,**d**,**f**) *MT-BCS* reconstructions when the distance of the scatterers from the origin is equal to (**a**,**b**) $d=d_{\min}=0.11$ [λ] (${\hat{\tau }}_{\max}=0.73$), (**c**,**d**) d=0.54 [λ] (${\hat{\tau }}_{\max}=0.83$), and (**e**,**f**) $d=d_{\max}=0.97$ [λ] (${\hat{\tau }}_{\max}=0.84$).

**Figure 9:**
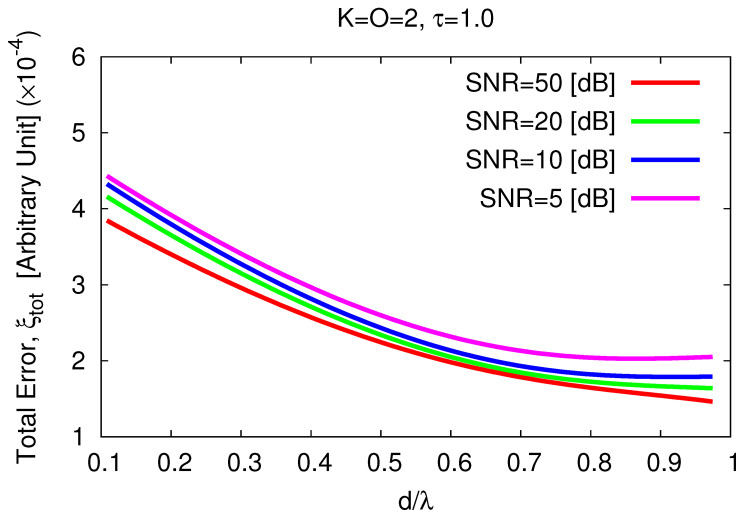
*Numerical Assessment* (K=2, O=2, τ=1.0, $SNR\in \left[5,\;50\right]$ [dB])—Behavior of the total error, $\xi_{tot}$, versus the distance of the scatterers from the origin, *d*.

Some representative test cases are reported in Figure 8: $d=d_{\min}=\ell \frac{\sqrt{3}}{2}=0.11$ [λ] (Figure 8a,b), d=0.54 [λ] (Figure 8c,d), and $d=d_{\max}=\left(L-\ell \right)\frac{\sqrt{3}}{2}=0.97$ [λ] (Figure 8e,f). As expected, the reconstruction worsens when the two objects get closer (Figure 9) until some artifacts appear when $d=d_{\min}$ [$\frac{{\left.\xi_{tot}\right\rfloor }_{d=0.11\;[\lambda ]}^{SNR=10\;[\mathrm{dB}]}}{{\left.\xi_{tot}\right\rfloor }_{d=0.97\;[\lambda ]}^{SNR=10\;[\mathrm{dB}]}}≃2.41$—Figure 8b vs. Figure 8f].

**Figure 10:**
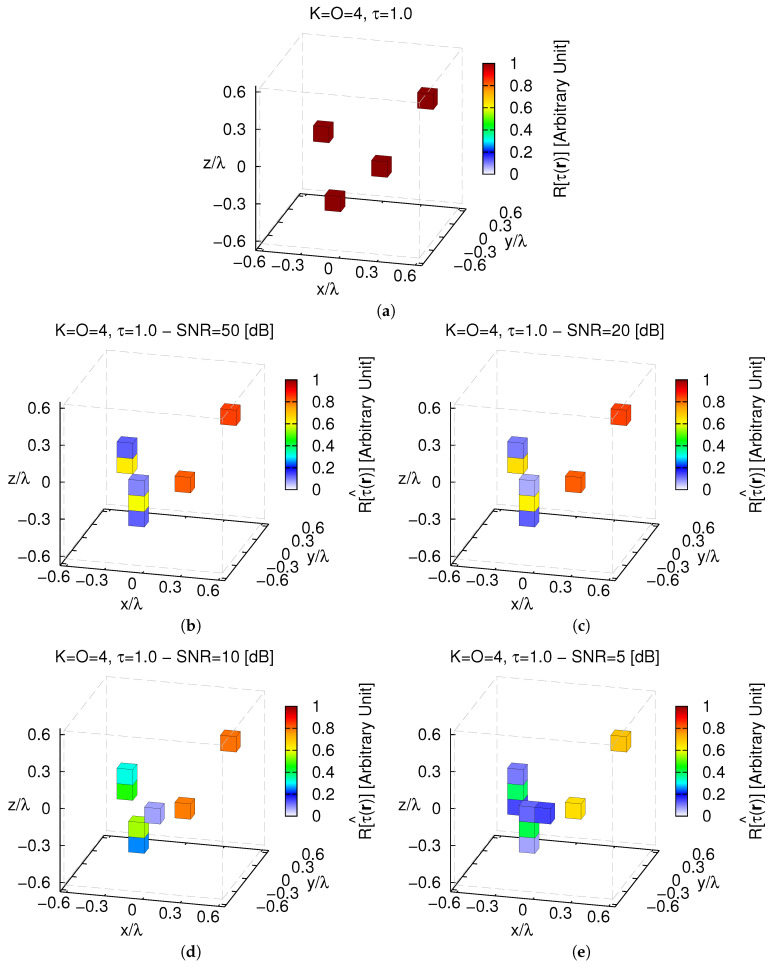
*Numerical Assessment* (K=4, O=4, τ=1.0)—(**a**) Actual contrast function and *MT-BCS* reconstructions when processing the scattering data characterized by (**b**) SNR=50 [dB] (${\hat{\tau }}_{\max}=0.84$), (**c**) SNR=20 [dB] (${\hat{\tau }}_{\max}=0.84$), (**d**) SNR=10 [dB] (${\hat{\tau }}_{\max}=0.80$), and (**e**) SNR=5 [dB] (${\hat{\tau }}_{\max}=0.70$).

Further lowering the *sparseness* of the actual profile causes a decrement of the *CS* performance as proven by the results of the third test case when O=4 and the K=4 single-voxel scatterers are randomly placed in D (Figure 10a).

**Figure 11:**
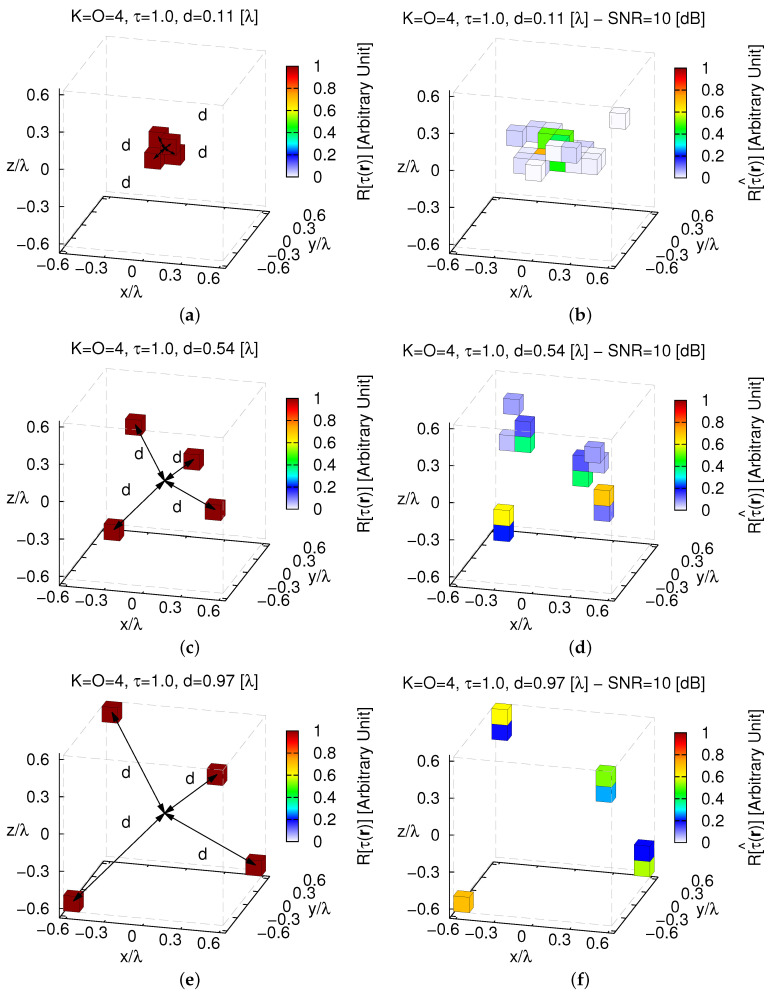
*Numerical Assessment* (K=4, O=4, τ=1.0, SNR=10 [dB])—(**a**,**c**,**e**) Actual contrast function and (**b**,**d**,**f**) *MT-BCS* reconstructions when the objects distance from the origin is (**a**,**b**) $d=d_{\min}=0.11$ [λ] (${\hat{\tau }}_{\max}=0.73$), (**c**,**d**) d=0.54 [λ] (${\hat{\tau }}_{\max}=0.70$), and (**e**,**f**) $d=d_{\max}=0.97$ [λ] (${\hat{\tau }}_{\max}=0.71$).

Unavoidably, the *MT-BCS* gets worse and undesired artifacts occur also when processing low-noisy data (e.g., ${\left.\xi_{tot}\right\rfloor }_{SNR=50\;[\mathrm{dB}]}=2.37\times 10^{-3}$—Figure 10b), but still without preventing the possibility to correctly identify K=4 separate objects (Figure 10b–e). In addition, in this case, changing the distance *d* of the objects with respect to the origin influences the quality of the retrieval as pictorially shown in Figure 11 (SNR=10 [dB]).

**Figure 12:**
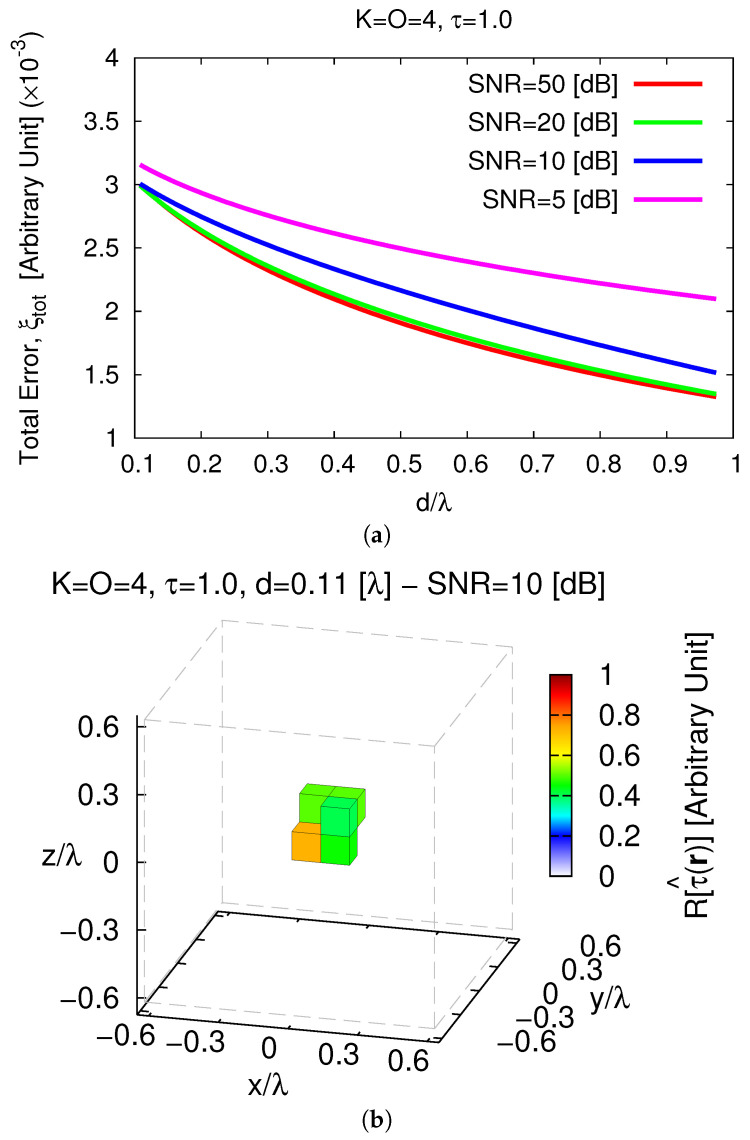
*Numerical Assessment* (K=4, O=4, τ=1.0, $SNR\in \left[5,\;50\right]$ [dB])—(**a**) Behavior of the total error, $\xi_{tot}$, as a function of the objects distance from the origin, *d* and (**b**) filtered ($\tau_{th}=10^{-3}$) *MT-BCS* reconstruction when the objects distance from the origin is $d=d_{\min}=0.11$ [λ] (${\hat{\tau }}_{\max}=0.73$).

Indeed, it turns out that more accurate images of D are yielded when the scatterers are far (i.e., d↑ ⇒ $\xi_{tot}↓$) as confirmed by the plot of $\xi_{tot}$ vs. *d* (Figure 12a). However, it cannot be neglected that a simple filtering (Figure 12b—$\tau_{th}=10^{-3}$) allows one to clearly resolve the scatterer support even in the most critical case (Figure 11a).

**Figure 13:**
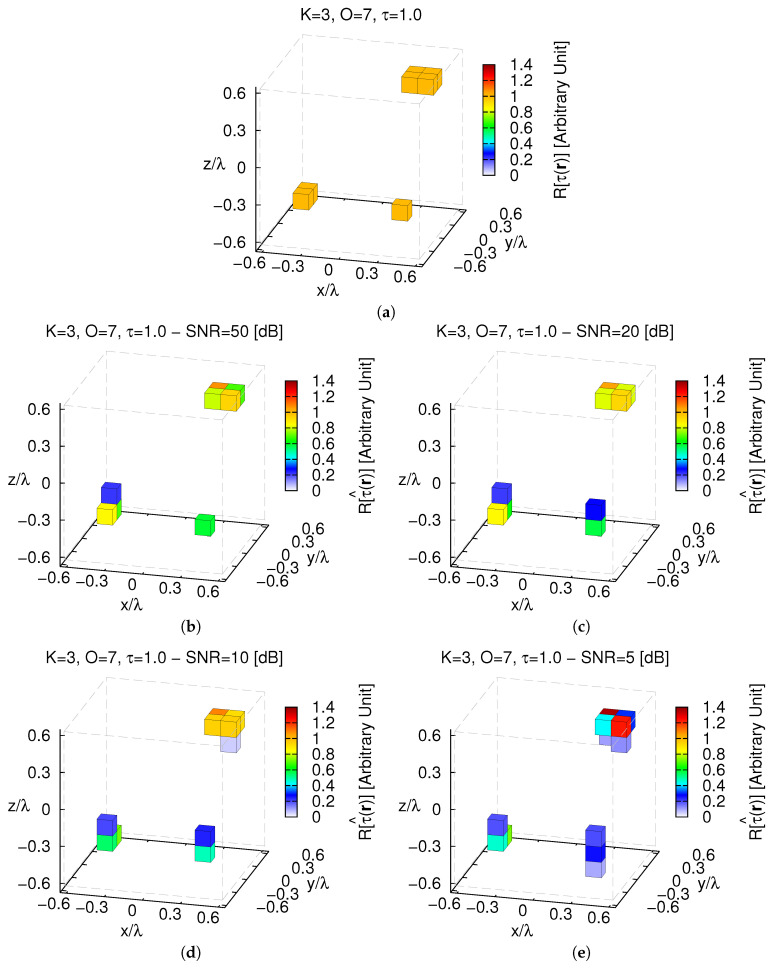
*Numerical Assessment* (K=3, O=7, τ=1.0)—(**a**) Actual contrast function and *MT-BCS* reconstructions when (**b**) SNR=50 [dB] (${\hat{\tau }}_{\max}=1.08$); (**c**) SNR=20 [dB] (${\hat{\tau }}_{\max}=1.05$); (**d**) SNR=10 [dB] (${\hat{\tau }}_{\max}=1.09$); and (**e**) SNR=5 [dB] (${\hat{\tau }}_{\max}=1.37$).

The next numerical test is devoted to validate the *MT-BCS* in a more complex *3D* imaging scenario concerned with non-uniformly shaped scatterers. More specifically, K=3 objects sized $\ell_{x}^{1}=\ell_{y}^{1}=\ell_{z}^{1}=0.125$ [λ], $\left[\ell_{x}^{2},\;\ell_{y}^{2},\;\ell_{z}^{2}\right]=\left[0.125,\;0.25,\;0.125\right]$ [λ], and $\left[\ell_{x}^{3},\;\ell_{y}^{3},\;\ell_{z}^{3}\right]=\left[0.25,\;0.25,\;0.125\right]$ [λ] (O=7) with contrast τ=1.0 have been imaged (Figure 13a).

**Figure 14:**
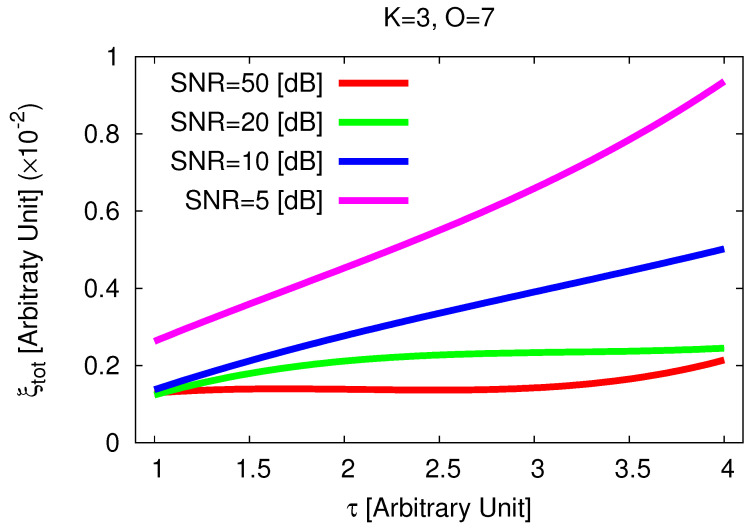
*Numerical Assessment* (K=3, O=7, $SNR\in \left[5,\;50\right]$ [dB])—Behavior of the total reconstruction error ($\xi_{tot}$) when processing the scattering data with the *MT-BCS*.

Despite the increased complexity and the reduced intrinsic-sparsity order of the scattering configuration, faithful reconstructions of the *3D* contrast distribution are yielded (Figure 13b–e) even though there is an over-estimation of the scatterers supports when highly-blurred data are at hand (e.g., Figure 13b vs. Figure 13e) and, consequently, an increase of the reconstruction error with the noise level (e.g., $\frac{{\left.\xi_{tot}\right\rfloor }^{SNR=5\;[\mathrm{dB}]}}{{\left.\xi_{tot}\right\rfloor }^{SNR=50\;[\mathrm{dB}]}}\approx 2.04$). Moreover, the previous considerations regarding the fidelity of the proposed strategy with respect to the target contrast are confirmed also when dealing with non-uniformly shaped scatterers (Figure 14).

**Figure 15:**
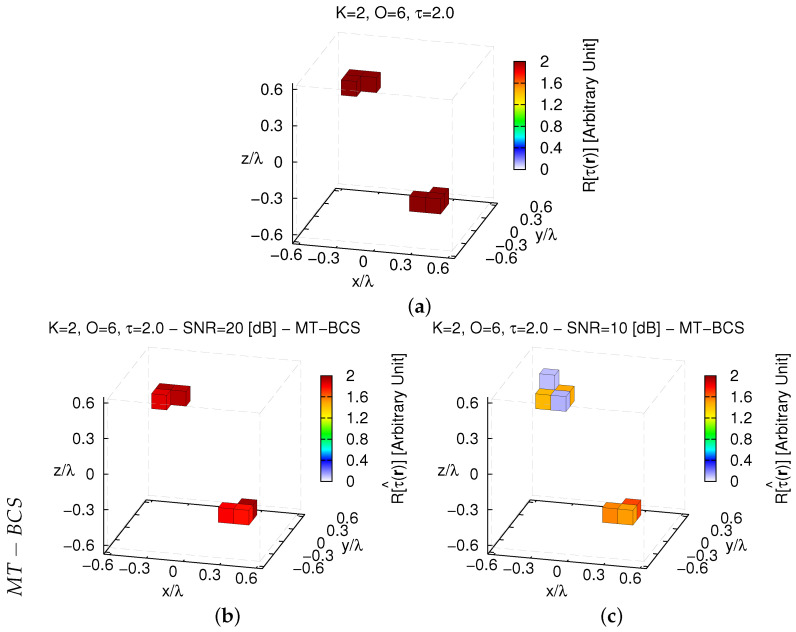

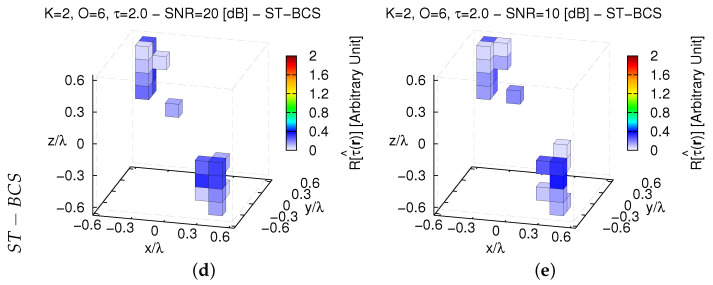
*Comparative Assessment* (K=2, O=6, τ=2.0)—(**a**) Actual contrast function and retrieved solutions by the (**b**,**c**) *MT-BCS* and (**d**,**e**) *ST-BCS* when processing noisy data at (**b**,**d**) SNR=20 [dB] (${\hat{\tau }}_{\max}^{MT-BCS}=1.96$, ${\hat{\tau }}_{\max}^{ST-BCS}=0.33$) and (**c**,**e**) SNR=10 [dB] (${\hat{\tau }}_{\max}^{MT-BCS}=1.68$, ${\hat{\tau }}_{\max}^{ST-BCS}=0.36$).

Finally, it is interesting to underline the advantage of using the *MT* extension of the *BCS*-based approach for solving the *3D*-*CSI* inversion problem instead of its “naive” single-task implementation (*ST-BCS* [23]) that does not impose any physical correlation in solving (14). Towards this end, let us consider the benchmark O=6 voxel arrangement in Figure 15a with two (K=2) “L-shaped” homogeneous (τ=2.0) objects.

**Figure 16:**
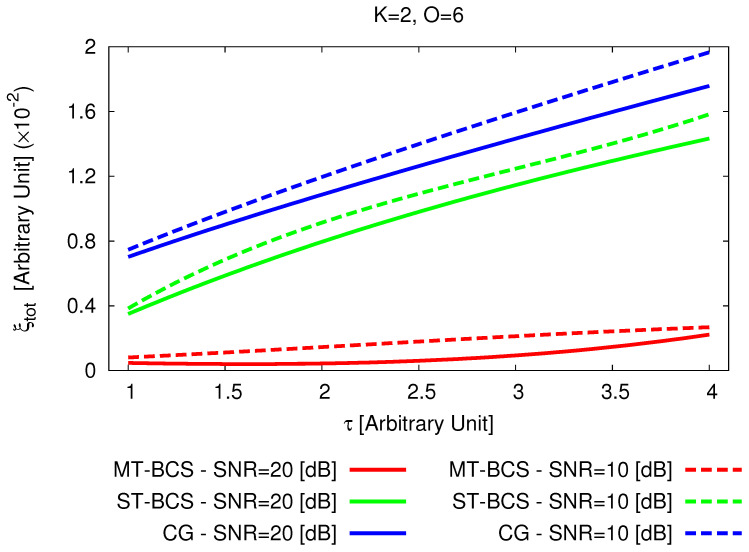
*Comparative Assessment* (K=2, O=6, $\tau \in \left[1.0,\;4.0\right]$, $SNR\in \left[10,\;20\right]$ [dB])—Behavior of the total error, $\xi_{tot}$, as a function of the object contrast, τ, when processing the scattering data with the *MT-BCS*, the *ST-BCS*, and the *CG* methods.

For illustrative purposes, the reconstructions with the *MT-BCS* (Figure 15b,c) and the *ST-BCS* (Figure 15d,e) when processing two different sets of noisy data [SNR=20 [dB]—Figure 15b,d; SNR=10 [dB]—Figure 15c,e] are shown. As it can be visually inferred, the *MT* strategy turns out to be more effective than the *ST* one in both locating and shaping the non-connected scattering regions as well as in estimating the actual contrast value. Such an inference is quantitatively confirmed by the error indexes since $\frac{{\left.\xi_{tot}\right\rfloor }_{ST-BCS}^{SNR=20\;[\mathrm{dB}]}}{{\left.\xi_{tot}\right\rfloor }_{MT-BCS}^{SNR=20\;[\mathrm{dB}]}}\approx 33.36$ (Figure 15b vs. Figure 15d) and $\frac{{\left.\xi_{tot}\right\rfloor }_{ST-BCS}^{SNR=10\;[\mathrm{dB}]}}{{\left.\xi_{tot}\right\rfloor }_{MT-BCS}^{SNR=10\;[\mathrm{dB}]}}\approx 7.04$ (Figure 15c vs. Figure 15e) (Table 3). Similar outcomes can also be drawn when changing the contrast of the scatterers as indicated by the behaviour of the reconstruction error $\xi_{tot}$ versus τ in Figure 16.

**Figure 17:**
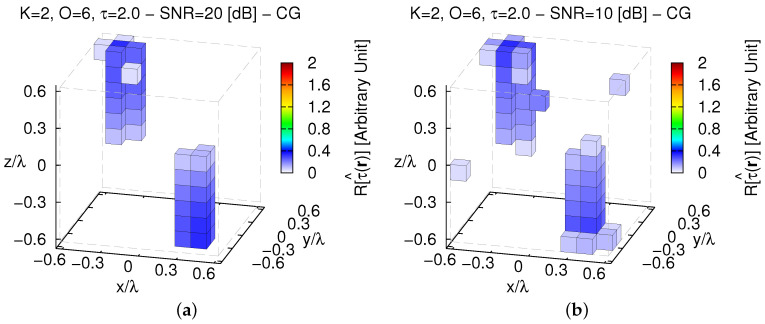
*Comparative Assessment* (K=2, O=6, τ=2.0)—*CG* reconstructions when processing noisy data characterized by (**a**) SNR=20 [dB] (${\hat{\tau }}_{\max}^{CG}=0.33$) and (**b**) SNR=10 [dB] (${\hat{\tau }}_{\max}^{CG}=0.36$).

To conclude the numerical assessment of the reconstruction capabilities of the *MT-BCS*, it has been compared with a competitive non-*CS* state-of-the-art approach. Towards this end, a deterministic *CG*-based inversion tool—still based on a *CSI* formulation of the scattering problem—has been applied to the same scenario in Figure 15a. The retrieved images of the dielectric profile of the investigation domain are shown in Figure 17a (SNR=20 [dB]) and Figure 17b (SNR=10 [dB]).

Without imposing *sparseness* priors, only the presence of K=2 scatterers lying in D can be deduced, but their contrasts are strongly under-estimated and their supports/shapes are unreliably predicted. Comparatively, the *MT-BCS* enables a reduction of the reconstruction error of about $\frac{{\left.\xi_{tot}\right\rfloor }_{CG}^{SNR=20\;[\mathrm{dB}]}}{{\left.\xi_{tot}\right\rfloor }_{MT-BCS}^{SNR=20\;[\mathrm{dB}]}}\approx 43.75$ (Figure 17a vs. Figure 15b—Table 3) and $\frac{{\left.\xi_{tot}\right\rfloor }_{CG}^{SNR=10\;[\mathrm{dB}]}}{{\left.\xi_{tot}\right\rfloor }_{MT-BCS}^{SNR=10\;[\mathrm{dB}]}}\approx 8.92$ (Figure 17b vs. Figure 15c—Table 3) times. Such conclusions are not limited to the contrast τ=1.0, but they also arise for stronger scatterers as detailed by the plot of $\xi_{tot}$ versus τ in Figure 16.

As for the computational efficiency, the *MT*-constrained exploitation of a sparseness promoting inversion technique allows a non-negligible reduction of the computational time, Δt, when processing *3D* scattering data (For the sake of fairness, all the computational times refer to non-optimized Matlab codes executed on a single-core laptop running at 2.20 GHz). Indeed, the *MT-BCS* is not only faster than the *ST-BCS*, thanks to the joint processing of the data (i.e., $\frac{{\left.\Delta t\right\rfloor }_{MT-BCS}}{{\left.\Delta t\right\rfloor }_{ST-BCS}}\approx 0.52$—Table 3), but it also overcomes the *CG* speed (i.e., $\frac{{\left.\Delta t\right\rfloor }_{MT-BCS}}{{\left.\Delta t\right\rfloor }_{CG}}\approx 0.032$—Table 3).

## 5. Conclusions

An innovative approach to efficiently solve the full-vectorial *3D-IS* problem has been presented. The retrieval of the volumetric contrast distribution of sparse non-weak scatterers has been tackled as a probabilistic *CSI*-based problem, which has been efficiently solved through a customized *MT-BCS* approach. The numerical analysis has pointed out the following key features of the proposed technique:

- Reliable *3D* reconstructions of the *EM* properties of the imaged domain are yielded processing scattering data also blurred with a non-negligible amount of additive noise;
- The inversion accuracy of the proposed *CS*-based approach depends on the degree of *sparseness* of the actual scenario with respect to the expansion basis at hand. However, it can be fruitfully and profitably applied when other/different (non-voxel) representations of the contrast source/contrast function are chosen [46];
- The *MT* implementation of the *BCS*-based inversion remarkably overcomes its single-task (*ST-BCS*) counterpart thanks to the profitable exploitation of the existing correlations between the *V* views and the scattered field components;
- The *MT-BCS* positively compares with other state-of-the-art approaches, also deterministic and non-*CS*, in terms of both reconstruction accuracy and computational efficiency.

Moreover, the methodological advancements of this work with respect to the state-of-the-art on the topic [47,48] include (*i*) the generalization of the *MT-BCS* strategy to handle *3D-IS* problems, differently from previous customizations of such an inversion paradigm which deal only with two-dimensional formulations [48], (*ii*) the derivation of a *BCS*-based imaging approach able to retrieve *3D* target contrast information, unlike state-of-the-art *Bayesian CS* contributions only dealing with the reconstruction of *equivalent sources* [47], and (*iii*) the analysis and validation of suitable operative guidelines for the optimal setting of the key calibration parameters of the introduced methodology. Future works will be aimed at extending the capabilities of the proposed approach to effectively deal with non voxel-sparse targets as well as with other applicative scenarios of great interest (e.g., subsurface imaging) including the processing of multi-frequency data [55].

## Figures and Tables

**Table 1:** *Numerical Assessment* (K=1, O=1, τ=1.0, W=100, *MT-BCS*)—Total error statistics.

SNR [dB]	$\xi_{\mathrm{tot}}^{\min}$	$\xi_{\mathrm{tot}}^{\max}$	$\xi_{\mathrm{tot}}^{\mathrm{avg}}$	$\xi_{\mathrm{tot}}^{\mathrm{var}}$
50	$7.99\times 10^{-5}$	$9.69\times 10^{-5}$	$9.10\times 10^{-5}$	$1.11\times 10^{-11}$
20	$8.37\times 10^{-5}$	$9.75\times 10^{-5}$	$9.12\times 10^{-5}$	$1.21\times 10^{-11}$
10	$8.66\times 10^{-5}$	$9.86\times 10^{-5}$	$9.27\times 10^{-5}$	$1.87\times 10^{-11}$
5	$8.68\times 10^{-5}$	$1.21\times 10^{-4}$	$9.78\times 10^{-5}$	$4.66\times 10^{-11}$

**Table 2:** *Numerical Assessment* (K=2, O=2, τ=1.0, W=100, *MT-BCS*)—Total error statistics.

SNR [dB]	$\xi_{\mathrm{tot}}^{\min}$	$\xi_{\mathrm{tot}}^{\max}$	$\xi_{\mathrm{tot}}^{\mathrm{avg}}$	$\xi_{\mathrm{tot}}^{\mathrm{var}}$
50	$1.49\times 10^{-4}$	$2.57\times 10^{-3}$	$4.60\times 10^{-4}$	$4.50\times 10^{-7}$
20	$1.56\times 10^{-4}$	$2.59\times 10^{-3}$	$4.63\times 10^{-4}$	$4.96\times 10^{-7}$
10	$1.61\times 10^{-4}$	$2.62\times 10^{-3}$	$4.72\times 10^{-4}$	$5.20\times 10^{-7}$
5	$1.83\times 10^{-4}$	$2.63\times 10^{-3}$	$5.30\times 10^{-4}$	$5.27\times 10^{-7}$

**Table 3:** *Comparative Assessment* (K=2, O=6, τ=2.0, $SNR\in \left[10,\;20\right]$ [dB])—Inversion performance indexes.

*Method*	SNR=20 [dB]			SNR=10 [dB]			Δt
	$\xi_{\mathrm{tot}}$	$\xi_{\mathrm{int}}$	$\xi_{\mathrm{ext}}$	$\xi_{\mathrm{tot}}$	$\xi_{\mathrm{int}}$	$\xi_{\mathrm{ext}}$	[s]
MT-BCS	$2.56\times 10^{-4}$	$4.07\times 10^{-2}$	0.00	$1.39\times 10^{-3}$	$1.53\times 10^{-1}$	$3.94\times 10^{-4}$	15.12
ST-BCS	$8.54\times 10^{-3}$	$6.28\times 10^{-1}$	$2.95\times 10^{-3}$	$9.79\times 10^{-3}$	$8.16\times 10^{-1}$	$3.06\times 10^{-3}$	29.08
CG	$1.12\times 10^{-2}$	$5.78\times 10^{-1}$	$7.79\times 10^{-3}$	$1.24\times 10^{-2}$	$5.91\times 10^{-1}$	$8.92\times 10^{-3}$	$4.66\times 10^{2}$
